# Lighting the way or casting new shadows? A One Health perspective on light-based technologies against clinically important antimicrobial-resistant bacteria

**DOI:** 10.3389/fcimb.2026.1867987

**Published:** 2026-07-16

**Authors:** Fábio P. Sellera, Fernanda V. Cabral, Martha S. Ribeiro

**Affiliations:** 1 School of Veterinary Medicine, Metropolitan University of Santos, Santos, São Paulo, Brazil; 2 Department of Internal Medicine, School of Veterinary Medicine and Animal Science, University of São Paulo, São Paulo, São Paulo, Brazil; 3 Division of Immunology and Molecular Medicine, Department of Molecular and Cell Biology, University of California, Berkeley, Berkeley, CA, United States; 4 Center for Lasers and Applications, Nuclear and Energy Research Institute (IPEN-CNEN), São Paulo, São Paulo, Brazil

**Keywords:** antimicrobial blue light (aBL), antimicrobial photodynamic therapy (aPDT), multidrug-resistant pathogens, photomedicine, ultraviolet irradiation (UV)

## Introduction

Antimicrobial resistance (AMR) is widely recognized as one of the most pressing global health challenges of the 21st century, contributing to increased morbidity and mortality, prolonged hospitalizations, and escalating healthcare costs. At the same time, the development of new antimicrobial agents has slowed considerably, while existing drugs continue to lose effectiveness due to the remarkable adaptability of pathogenic microorganisms. This imbalance has intensified the search for alternative, non-antibiotic strategies to control infections without exacerbating antibiotic resistance (Naghavi et al., 2024).

Traditionally, AMR has been approached primarily within the boundaries of human medicine. However, this perspective is no longer sufficient. The emergence and dissemination of multidrug-resistant (MDR) organisms are now understood as a One Health issue, driven by the interconnected dynamics among humans, animals, and the environment (Al-Khalaifah et al., 2025). Human-associated clinically relevant pathogens, including members of the ESKAPEE group (*i.e.*, *Enterococcus faecium*, *Staphylococcus aureus*, *Klebsiella pneumoniae*, *Acinetobacter baumannii*, *Pseudomonas aeruginosa*, *Enterobacter* spp., and *Escherichia coli*), circulate across these domains, colonizing or infecting food-producing animals, companion animals, wildlife, as well as contaminating environmental sources such as soil and water systems impacted by anthropogenic activity (Khasapane et al., 2024). Many of these organisms are classified as World Health Organization (WHO) priority pathogens, a designation based on factors such as limited therapeutic options, high mortality rates, healthcare burden, and their capacity to acquire and disseminate resistance (World Health Organization, 2024). In this context, interventions that fail to consider these interconnections risk offering only partial or temporary solutions.

Light-based antimicrobial technologies, such as antimicrobial photodynamic therapy (aPDT), antimicrobial blue light (aBL), and ultraviolet (UV) irradiation, have emerged as promising alternatives or adjuncts to conventional antimicrobial approaches. These strategies rely on physical and photochemical mechanisms rather than traditional biochemical targets, which reduces the likelihood of classical resistance development. In aPDT, a light-activated photosensitizer (PS) generates reactive oxygen species (ROS) that damage multiple cellular targets. In aBL, endogenous chromophores in microorganisms are excited, producing cytotoxic oxidative stress. UV irradiation, particularly in the UVC range, induces direct DNA damage, impairing replication and viability (Sabino et al., 2020).

We argue that, while these technologies hold significant promise, their development and implementation remain fragmented across disciplines. Without a coherent One Health framework, light-based antimicrobial strategies risk being applied in isolated contexts, limiting their broader impact and potentially altering microbial communities and ecological balance in unintended ways.

## From bench to bedside: are light-based strategies ready for mainstream human healthcare?

In human medicine and dentistry, clinical translation of aPDT has focused primarily on localized infections, including chronic wounds, diabetic foot ulcers, oral infections, and dermatological conditions, using laser-, LED-, and more recently OLED-based platforms (Grandi et al., 2018; Brandão et al., 2023; Jao et al., 2023; Alves et al., 2025; Piksa et al., 2023). Likewise, aBL has gained increasing attention as a photosensitizer-free antimicrobial platform, with growing evidence supporting its activity against diverse pathogens (Leanse et al., 2022) and its potential applications in recurrent infections (Shalaby et al., 2024), although current clinical evidence remains limited to acne treatment (Diogo et al., 2021). Their application in topical infections demonstrates their versatility, especially against biofilm-associated and MDR pathogens. These approaches are particularly attractive in scenarios where conventional antibiotics fail or where biofilm tolerance significantly reduces drug efficacy. Importantly, both approaches are often used as adjuvant therapies, as they exhibit synergistic effects when combined with other treatments and/or antimicrobials.

Despite these advances, the transition from bench to bedside in antimicrobial applications remains incomplete (Sellera et al., 2022). Clinical adoption is hindered by the limited number of well-designed randomized controlled trials specifically targeting infectious diseases, particularly those caused by WHO priority pathogens, and by the lack of harmonized frameworks to guide treatment parameters, including PS type and concentration (for aPDT), as well as light wavelength, fluence rate, and exposure time (for both aBL and aPDT). However, framing protocol variability solely as a weakness may be misleading. Given the diversity of infections, tissues, and host conditions, rigid standardization may be neither feasible nor desirable. Rather than strict standardization, the field may benefit from developing adaptable, evidence-based guidelines that accommodate biological and clinical heterogeneity while enabling reproducibility and regulatory acceptance.

UV-based technologies, especially for environmental decontamination in healthcare settings, have demonstrated clear benefits in reducing microbial burden on surfaces and in the air (Scott et al., 2022). However, their implementation is often constrained by safety concerns related to human exposure, logistical challenges, and infrastructure requirements. Emerging approaches, such as far-UVC irradiation, have shown promising results for continuous environmental disinfection, including effective inactivation of airborne pathogens under room-scale conditions (Eadie et al., 2022). However, further studies are still needed to fully establish their long-term safety, implementation strategies, and real-world effectiveness.

Taken together, these observations suggest that light-based antimicrobial technologies are not yet fully ready for widespread clinical integration. More importantly, broader integration with veterinary and environmental contexts remains essential to maximize their contribution to AMR mitigation strategies.

## Promise and gaps in veterinary applications

In veterinary medicine, the application of light-based antimicrobial technologies is considerably less developed. While aPDT has been explored in companion animals, livestock, and occasionally wildlife, the available evidence remains limited and often relies on case reports, small cohorts, or studies lacking appropriate controls (Sellera and Ribeiro, 2026).

Despite these limitations, several veterinary applications have yielded encouraging clinical outcomes. In companion animals, aPDT has been successfully used to manage localized infections caused by both bacterial and fungal pathogens, including canine otitis externa associated with carbapenem-resistant *P. aeruginosa*, dermatophytosis caused by *Microsporum canis*, and feline sporotrichosis (Sellera et al., 2019; Cabral et al., 2021, 2022). In food-producing animals, favorable results have been reported for bovine digital dermatitis associated with pathogenic spirochetes, and for caseous lymphadenitis in sheep (Sellera et al., 2021, 2016). Applications have also been described in wildlife and exotic species, including pododermatitis in penguins and infectious stomatitis in captive snakes (Nascimento et al., 2015; Grego et al., 2017). These studies demonstrate the versatility of aPDT across diverse host species and infectious conditions.

Importantly, several of the pathogens targeted in these studies are recognized One Health threats and major clinical challenges in veterinary medicine due to their capacity to circulate across animal, human, and environmental compartments, emphasizing the potential role of light-based technologies within One Health-oriented antimicrobial strategies.

Clinical evidence for aBL in veterinary settings remains even more scarce than that available for aPDT. Most available studies focus on *in vitro* antimicrobial activity against isolates obtained from animal infections, with a notable lack of well-designed *in vivo* or clinical investigations (Schnedeker et al., 2017; Dos Anjos et al., 2019, 2020; Gigante et al., 2024; Ngowwatana et al., 2025). As a result, robust clinical recommendations remain scarce.

The intrinsic diversity of veterinary practice presents unique challenges. Differences in species, anatomy, physiology, and husbandry systems complicate the standardization of treatment protocols and the design of large-scale clinical trials. Unlike human medicine, where patient populations are relatively homogeneous, veterinary applications must account for a wide range of biological and environmental variables.

Nevertheless, this complexity should be viewed not solely as a barrier but also as an opportunity. Veterinary settings represent critical nodes in the transmission of AMR, particularly within food production systems and companion animal-human interfaces. The integration of light-based technologies into these contexts could reduce antimicrobial use, mitigate the dissemination of resistance, and provide alternative treatment options for refractory infections.

At present, significant knowledge gaps persist across species and clinical indications. Few studies have evaluated long-term outcomes, recurrence rates, reductions in antimicrobial consumption, or the impact of these interventions on the carriage and transmission of antimicrobial-resistant microorganisms. Furthermore, evidence from randomized controlled trials and multicenter investigations remains exceptionally limited, restricting the development of evidence-based guidelines and regulatory pathways for clinical adoption.

Moreover, current research efforts remain insufficiently integrated and underfunded, often disconnected from both human healthcare and environmental considerations. Without coordinated, species-specific, and context-aware investigations, the potential of these technologies within veterinary medicine will remain largely untapped.

## Environment: disinfection without ecological consequences?

Within the environmental dimension of One Health, UV irradiation is the most established light-based antimicrobial strategy. Its application in water treatment, wastewater management, and surface disinfection offers clear advantages, including effective microbial inactivation without the chemical residues associated with traditional disinfectants (Albertini et al., 2023). These characteristics position UV technologies as attractive tools for reducing environmental reservoirs of pathogenic microorganisms.

However, this perspective warrants careful scrutiny. Environmental microbial communities play essential roles in ecosystem functioning, including nutrient cycling, biodegradation, and maintenance of ecological balance. Broad-spectrum disinfection strategies, even when non-chemical, do not discriminate between pathogenic and beneficial microorganisms. As a result, their widespread use may disrupt microbial ecosystems, leading to unintended consequences that remain poorly understood.

Moreover, while UV irradiation effectively inactivates microorganisms, it does not necessarily eliminate extracellular DNA or resistance genes from the environment. These genetic elements may persist and contribute to horizontal gene transfer, sustaining the spread of AMR even in the absence of viable cells. Additionally, selective pressures imposed by repeated exposure to oxidative or radiation-based stressors may favor the emergence of more tolerant microbial populations, raising questions about long-term ecological impacts.

Operational challenges further complicate implementation. Factors such as turbidity, organic load, and shadowing effects can reduce the efficacy of UV systems in complex environmental matrices. Energy consumption, infrastructure requirements, and cost may also limit accessibility, particularly in low-resource settings where AMR burden is often highest.

Therefore, while environmental applications of light-based technologies are promising, they should not be assumed to be inherently benign. A One Health perspective requires that their ecological impact be evaluated alongside their antimicrobial efficacy.

## Toward an integrated One Health framework

A central limitation in the current development of light-based antimicrobial technologies is the lack of integration across human, animal, and environmental domains. These technologies are typically investigated within disciplinary silos, with little consideration of how their application in one context may influence outcomes in another.

This fragmented approach contrasts with the interconnected nature of AMR transmission. For example, reducing antimicrobial use in veterinary medicine through alternative therapies may decrease the dissemination of antimicrobial-resistant bacteria into the food chain and environment. Similarly, environmental disinfection strategies may influence microbial communities, ultimately affecting both human and animal health. Conversely, poorly designed interventions in any single domain may inadvertently exacerbate resistance dynamics elsewhere.

We contend that light-based antimicrobial technologies should be reconceptualized not merely as alternative treatments, but as ecological interventions operating within complex, interconnected systems. This shift in perspective has several implications. First, it underscores the need for harmonized reporting frameworks that enable comparison across studies and disciplines. Second, it highlights the importance of interdisciplinary collaboration, bringing together expertise from microbiology, photomedicine, veterinary science, environmental science, and public health. Third, it calls for the incorporation of ecological risk assessment into the development and deployment of these technologies.

## Final remarks

Light-based antimicrobial technologies represent a promising addition to the arsenal against AMR, offering mechanisms of action that differ fundamentally from those of conventional antibiotics and reducing the likelihood of classical resistance. However, their current trajectory, characterized by fragmented research efforts and context-specific applications, limits their potential impact.

A One Health framework provides a necessary lens through which these technologies can be developed and implemented more effectively. This requires moving beyond isolated applications toward integrated strategies that account for the dynamic interactions among humans, animals, and the environment. It also demands a critical evaluation of both benefits and risks, including potential ecological consequences and long-term effects on microbial communities.

An important remaining challenge lies in the limited mechanistic understanding of light-based antimicrobial therapies *in vivo*. Current research has largely focused on demonstrating clinical outcomes, such as reductions in microbial burden and improvements in wound healing. However, comparatively few studies have investigated how these approaches interact with host biology, particularly in terms of immune modulation. This gap is critical, as the therapeutic impact of these technologies likely extends beyond direct microbial inactivation and involves complex host-pathogen interactions. Addressing this limitation will require stronger integration of basic and translational research, moving beyond parameter optimization to a more comprehensive understanding of the underlying biological mechanisms.

Furthermore, the predominantly topical nature of current applications restricts their use to localized infections, limiting their relevance for systemic conditions such as pneumonia, urinary tract infections, or sepsis. This highlights a critical domain in which conventional antibiotics continue to outperform light-based approaches, owing to their systemic reach and well-established pharmacokinetics. Overcoming these barriers remains a key challenge for broader clinical adoption, and expanding light-based technologies to deep-seated or disseminated infections is an emerging frontier (Silva et al., 2025). In this context, light dosimetry plays a central role, as wavelength, irradiance, fluence, exposure time, and light delivery determine whether an effective optical dose can be achieved at the site of infection. In addition, successful translation requires optimization of PS-related parameters, including biodistribution and interactions with microbial targets. Strategies such as improved PS delivery platforms, systemic administration of selected PSs, and advanced light delivery approaches, including endoscopic illumination and large-field/whole-body systems, may broaden their applicability beyond superficial infections.

Ultimately, the question is not only whether light-based antimicrobial technologies can help combat AMR, but how they should be deployed to do so responsibly. Without coordinated, interdisciplinary efforts and a commitment to ecological awareness, these technologies may illuminate new paths in antimicrobial control but also cast unintended shadows.
